# New evaluation index for the retainability of a swimmer’s horizontal posture

**DOI:** 10.1371/journal.pone.0177368

**Published:** 2017-05-09

**Authors:** Yasunori Watanabe, Kohji Wakayoshi, Teruo Nomura

**Affiliations:** 1 Faculty of Sports Science, Sendai University, Miyagi, Japan; 2 Faculty of Human Sciences, Osaka University of Economics, Osaka, Japan; 3 Graduate School of Science and Technology, Kyoto Institute of Technology, Kyoto, Japan; Universite de Nantes, FRANCE

## Abstract

This study aims to investigate the effect of changes in buoyancy when a swimmer respires in a horizontal posture. We attempted to evaluate the levelness of swimmers’ streamline posture by simultaneously measuring the lung capacity and buoyancy under water. The buoyancy was measured based on the changes in the vertical loads of the upper and lower limbs on the subjects’ streamline posture under water. The horizontal x-axis as lung ventilation and the vertical y-axis as buoyancy forms a linear equation y = ax + b. The relation between hand (upper-limb) buoyancy and lung ventilation is defined as y = a1x + b1 and that between foot (lower-limb) buoyancy and lung ventilation as y = a2x + b2. Horizontal levelness was calculated as a ratio by dividing a2 by a1 using the inclination (a) values from these formulas for an underwater streamline posture. We defined this ratio as the breathing–balance (BB) ratio. Although the performance levels in the present study did not show any difference in the absolute quantity of air that humans can inhale in a streamline posture, the BB ratio was higher in a statistically significant manner in junior swimmers competing at international levels compared with the other groups of subjects (P < 0.001). This statistical difference in horizontal levelness, despite the absence of a noticeable difference in the absolute quantity of inhaled air, may be attributable to the way in which each person inhales and exhales air. Top-level junior swimmers that exhibited a high BB ratio might have inhaled in a way that would counteract the sinking of the lower limbs, for example, through abdominal respiration. When exhaling, on the other hand, they might have let out air gradually to mitigate the acceleration force involved in submerging the lower limbs.

## Introduction

Many factors determine the swimming ability. In particular, buoyancy is very important. If, for instance, a swimmer is able to effectively utilize his or her buoyancy, the swimming performance will improve. Several reports have reported that buoyancy intervention, for instance, artificially adding buoyancy, positively influences the swimming performance in terms of factors such as swim time, energy cost, and drag force (Capelli et al. [1], Chatard et al. [2],Cordain & Kopriva. [3], Toussaint et al. [4], Zamparo et al. [5]). Recent studies have performed simulation analyses to review the influence that buoyancy torque has on the actual performance of swimmers (Yanai [6, 7], Nakashima [8]). Although these reports provide several interesting suggestions, none have conducted an actual survey of buoyancy.

While swimming, humans are constantly affected by two physical forces: gravity and buoyancy. There is generally a “gap” in a swimmer’s streamline posture at the point at which buoyancy takes over (commonly referred to as the “center of buoyancy,” CoB) and the point at which gravity takes over (the “center of mass”, CoM); the CoB is located near the swimmer’s head, and the CoM is located near the swimmer’s leg (Hay [9]). The further apart the CoB is from the CoM, the bigger the buoyancy torque will be, causing the swimmer’s lower limbs to sink. This sinking of the lower limbs is believed to increase the pressure resistance facing the swimmer by widening the angle of attack in relation to the direction of propulsion. This degrades the swimming performance. Previous studies that have investigated the position of the CoB have reported the effects that age, gender, physique, lung capacity, and posture have on buoyancy in relation to the CoB/CoM distance (Carmody [10], Carter [11, 12], Gagnon & Montpetit [13], Rork & Hellebrandt [14]). Gagnon and Montpetit [13], through a unique method, attempted to measure the buoyancy affecting the human body. However, the subjects were fixed on an aluminum table in an anatomically standard supine posture during measurement. This measurement method is different from the actual swimming posture. In particular, it cannot evaluate the ability of the swimmer to control posture while floating under water. Given that swimmers swim in a streamline posture, it is essential to have the subjects assume this posture while buoyancy is measured if we are to obtain useful insights into improving one’s swimming performance. McLean & Hinrichs [15] conducted buoyancy measurements when their test subject’s entire body was floating; hence, McLean & Hinrichs’ study is a more practical attempt toward examining a swimmer’s posture-retention ability. However, a part of the subject’s body came out of water during the measurement and was not submerged. To correctly evaluate the buoyancy acting on the body of a swimmer, the body must maintain being under the surface of water.

Cureton [16] describes the influence that breathing has on a human’s buoyancy, floating, and balance. The body’s cubic volume increases with inspiration, thus proportionally increasing the buoyancy. In contrast, during expiration, the body sinks as the volume of air filling the lungs decreases. In fact, there are people who can float in the horizontal posture for a long time, and there are people who cannot float for a long time as their legs sink immediately. One of the factors may be the difference in how buoyancy works. It has never been quantitatively investigated how much the horizontality of the streamline posture changes with an increase/decrease in buoyancy due to respiration. Evaluating the relationship between respiration and horizontality is meaningful because it is considered to contribute toward improving the swimming performance.

The purpose of this study is to examine the influence that buoyancy fluctuations (accompanying the breathing cycle) have on a swimmer’s streamline posture as well as to observe the chronological changes in the vertical load on both the swimmer’s wrists and ankles to propose a new index for simply and quantitatively evaluating a swimmer’s horizontal posture.

## Materials and methods

### Ethics statement

All experiments related to this study were approved by the Biwako Seikei Sport College Library and Science Committee (Human Subject Committee, Approval No. 68). Written, informed consents were obtained from all subjects prior to their participation. Researchers fully explained the purpose, methods, and related risks to all test subjects both verbally and in writing. The test subjects were all volunteers, and we confirmed that it was possible for them to discontinue participation at any time. For subjects under the age of 18, we obtained informed consents from their parents on their behalf. This study was conducted in accordance with the Declaration of Helsinki.

### Test subjects

Eight athletes who were involved in their respective colleges’ sports clubs but had never received specialized swimming instructions (one rugby player, five football (soccer) players, one basketball player, and one badminton player) comprised the non-swimmer (NS) group; their age was 18.8 ± 0.4 years. Likewise, eight competitive swimmers from the college’s swimming club comprised the collegiate swimmers (CS) group; their age was 19.0 ± 0.7 years. Finally, ten international competitive junior swimmers comprised the junior top swimmers (JTS) group; their age was 17.0 ± 0.8 years.

### CoM and CoB Measurements

**Fig 1:**
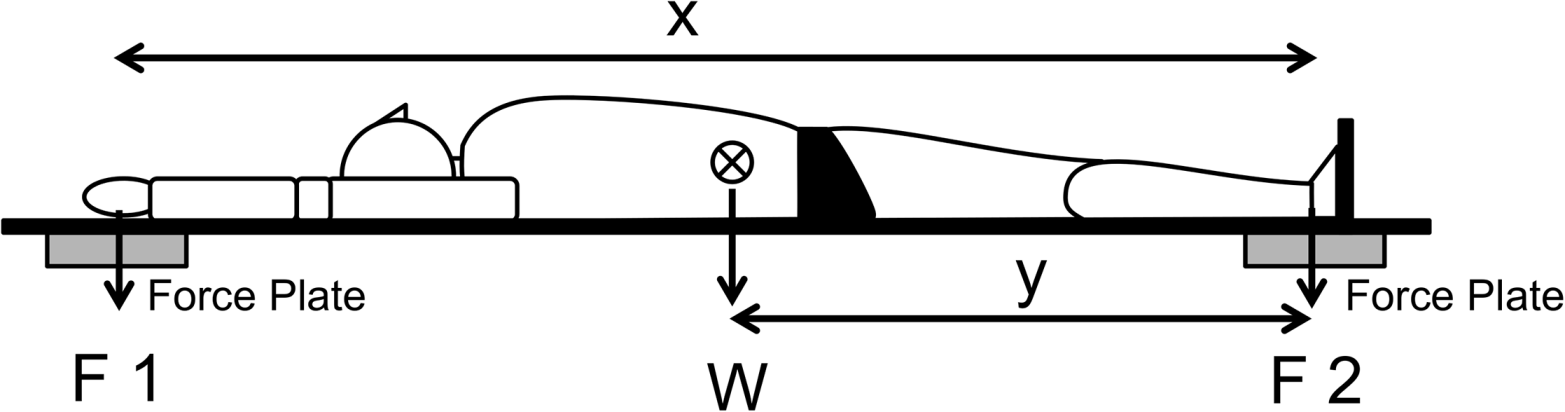
Overview of CoM Measurements. 10.6084/m9.figshare.2059968.

The CoM of each test subject was measured using the CoM Trembling Measuring System G-Gravity (4Assist, Inc.), which uses simplified force plates; furthermore, using the reaction board method (Hay [9]), see Fig 1, we were able to conduct our measurements on land. Each force plate was placed on the sides of the head and sides of the feet of the subjects. A board was placed on the force plates, and the subjects were positioned on it. Each test subject held his/her breath after inspiration and remained immobile for three seconds. While in the streamline posture, the distance from the subject’s foot (lateral malleolus) to the subject’s hand (center of the fist) was designated as x, and the distance from the subject’s foot to the subject’s CoM was designated as y. Calculations were made using the following equation, where the weight (F1_CoM_ + F2_CoM_) and force (F1 _CoM_) vertically act on the hand:

$$y={F1}_{CoM}∙x/({F1}_{CoM}+{F2}_{CoM})$$ (1)

**Fig 2:**
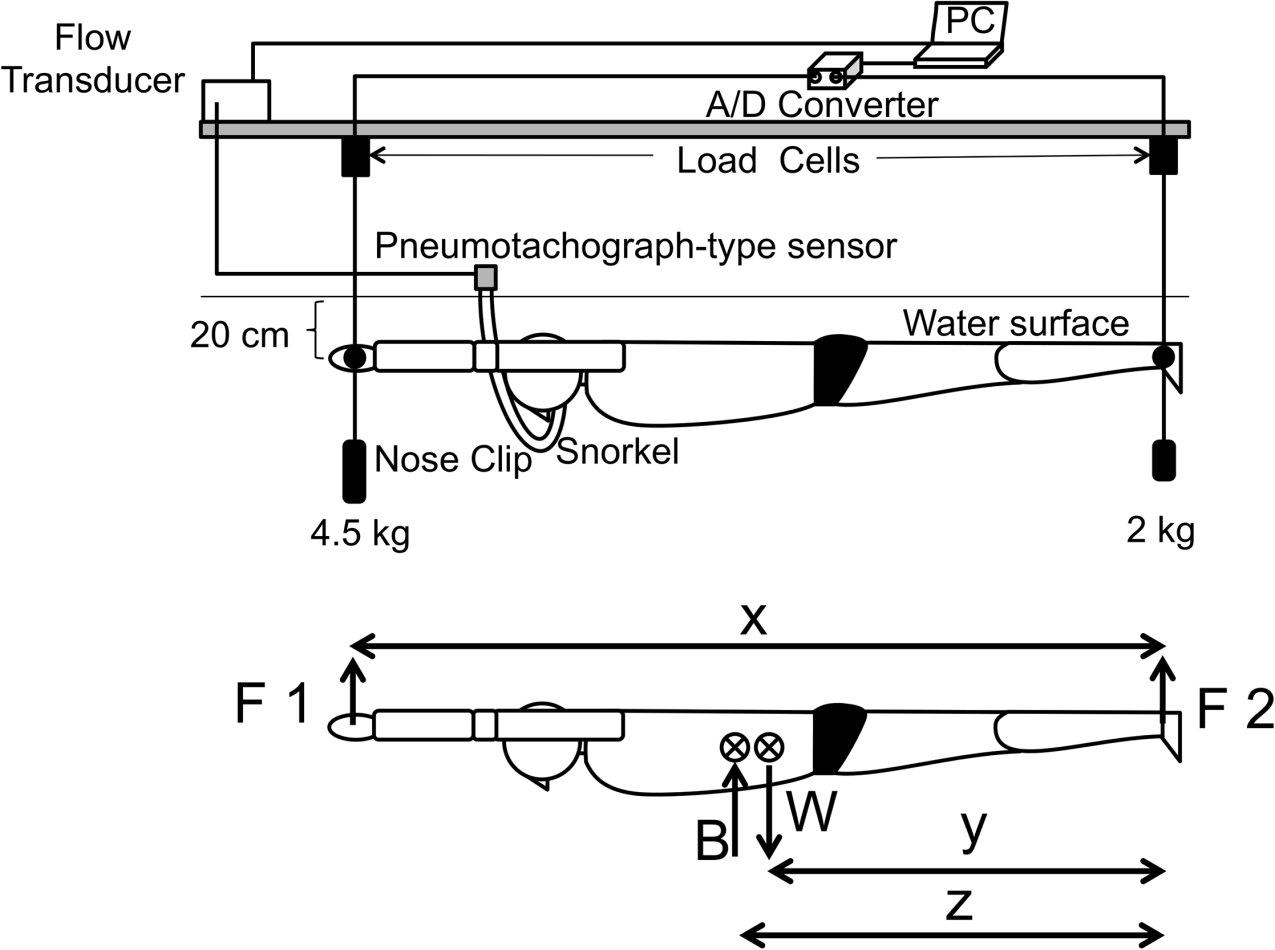
Apparatus Used for CoB Measurements and the associated free body diagram. 10.6084/m9.figshare.2059971.

The method of CoB measurement proposed by McLean & Hinrichs [17] was used for reference (see Fig 2). We installed a frame to secure the subject’s body on the poolside and attached Tension/Compression Load Cells (LUR-A-200NSA1, KYOWA ELECTRONIC INSTRUMENTS CO., LTD) on the hands and feet in a vertical direction to measure the force exerted on them in that direction. The sampled signals were amplified by a Digital Transducer Indicator (TD-250T, TAKEI KIKI KOGYO CO., LTD) and recorded by a computer. In all measurements, each subject was instructed to breathe through a snorkel and wear a nose clip to prevent air leakage from any other body part except the mouth. The tips of the snorkels were attached to pneumotachographic sensors (ARCO SYSTEM Inc.) and connected to a dedicated amplifier (FM-200XB, ARCO SYSTEM Inc.) to develop pressure differences. Because the difference is converted into velocity data and output by the amplifier, we converted these data digitally and recorded them using the computer. We calculated the flow volume by integrating the velocity data. The subjects’ bodies were completely submerged in water. Load cells were installed on both the vertical line passing through the center of the subject’s hand grip (F1) and the vertical line passing through the center of the subject’s ankles (F2). In addition, the hand grip and foot tether were both set at depths of 20 cm. We attached a 4.5-kg weight to the hand grip and a 2-kg weight to the foot tether so that the subject could remain in a stable streamline posture underwater (see Fig 2).

With the test subjects still underwater, we completely synchronized the data measured by both the ventilation flow meter and load cell. Calibrations were conducted with all experimental apparatuses in their set positions and with offset buoyancy acting on the weights attached to the hand grip and foot tether; the reference value was set to 0, and calibrations were performed in still water.

If we assume that a subject remains still in water, the buoyancy force (B) can be calculated as

$$B=-({F1}_{CoM}+{F2}_{CoM})-{F1}_{CoB}-{F2}_{CoB}.$$ (2)

The CoB from the foot area (z) can be calculated by inserting Eqs (1) and (2) into the following formula:

$$z=({(F1}_{CoB}∙x)+(({F1}_{CoM}+{F2}_{CoM})∙y))/\left({F1}_{CoM}+{F2}_{CoM}\right).$$ (3)

Hence, the CoB/CoM distance in a swimmer’s underwater horizontal posture (*d*) can be calculated as

d=z−y. (4)

The test subjects’ inspirations were limited to six breaths per min (5-s inspirations, 5-s expirations) using a metronome. We measured the data 20 s after the commencement of the examination because we had to wait for the person being tested to become static. Every measurement was conducted in still water.

### Horizontal-posture evaluation

When lung ventilation increases owing to inspiration, the vertical load on the hand/foot also increases; this establishes a linear relation between these two properties. Therefore, setting the horizontal x-axis as lung ventilation and the vertical y-axis as buoyancy forms a linear equation y = ax + b. The relation between hand (upper-limb) buoyancy and lung ventilation is defined as y = a1x + b1 and that between foot (lower-limb) buoyancy and lung ventilation as y = a2x + b2. Using the inclination (a) values from these formulas, the breathing–balance (BB) ratio, which shows the test subject’s horizontal retention rate within a streamline posture underwater, can be obtained:

$$BB\;Ratio=\frac{a2}{a1}.$$

### Torque calculations

**Fig 3:**
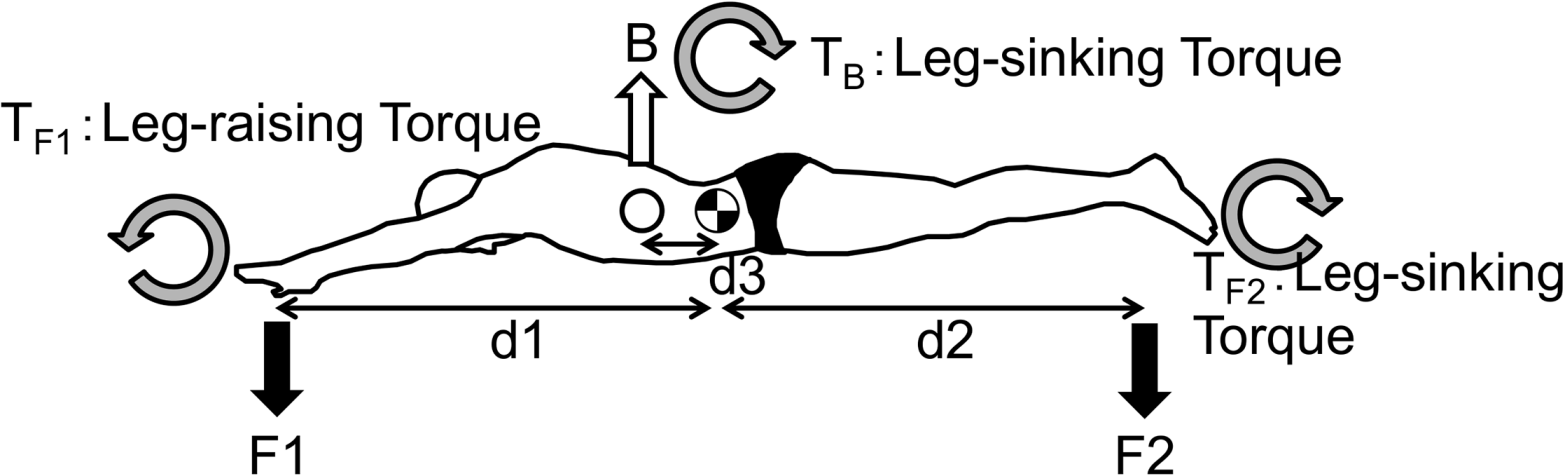
The Relation between the forces affecting a swimmer’s body and torque. 10.6084/m9.figshare.2059974.

Fig 3 schematically shows the forces affecting a swimmer’s body. Since torque (T) = F · d, the upper-limb torque (T _F1_), with the CoM as the center of rotation, can be gained from F1 · d1. Similarly, the lower-limb torque (T_F2_) can be gained from F2 · d2, and the buoyancy torque (T_B_) can be gained from B · d3. In this study, we defined T_F1_ as the leg-raising (positive) torque and T_F2_ and T_B_ as the leg-sinking (negative) torque. When the CoM is set as the rotational axis, if we assume that the torques on the right and left sides are in balance and retain the swimmer’s horizontal position, the difference between T_F1_ and T_B_ must be equal to T_F2_. Therefore, we have T_F1 −_ T_B_ = T_F2_.

### Statistics processing

To compare each measured item across three varying levels of swimming ability exhibited by our test subjects, a one-way analysis of variance (ANOVA, no correspondence) was employed. ANOVA tests were performed using the IBM SPSS v.19 statistics software. The significance level was set at a 0.05 critical rate.

## Results

**Fig 4:**
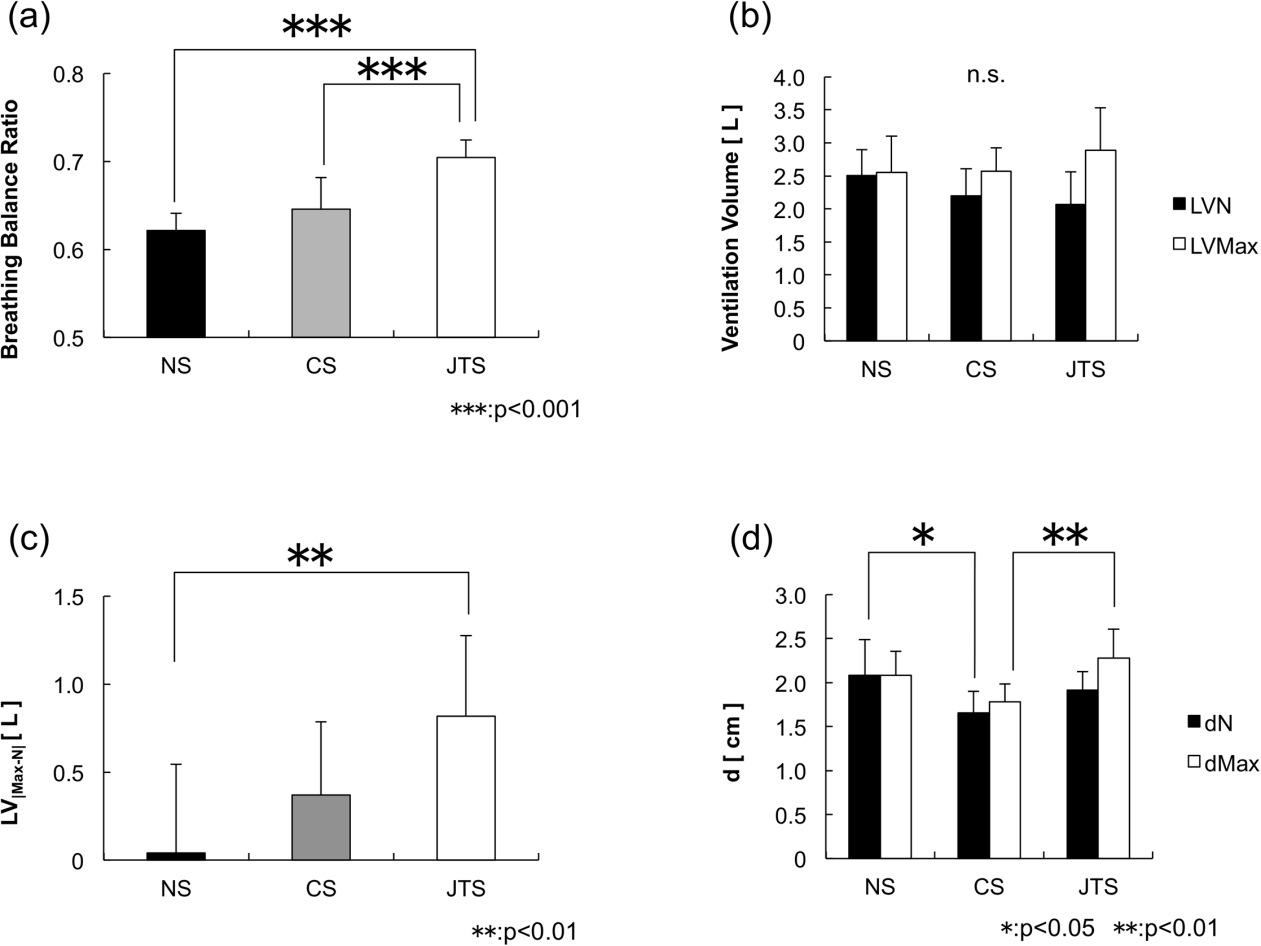
Comparison of the Breathing–Balance Ratio, lung ventilation, and CoB/CoM distance. (a) The BB ratio results show that the JTS group displayed a higher horizontal retention rate than the other groups (JTS group: 0.70 ± 0.02, CS group: 0.65 ± 0.04, NS group: 0.62 ± 0.02). (b) No difference in lung ventilation was detected among the three groups, either in neutral buoyancy ventilation (LV_N_) or in maximum buoyancy ventilation (LV_Max_). (c) The JTS group exhibited significantly greater lung ventilation variation (i.e., the difference between the neutral and maximum ventilation) than the NS group: 0.82 ± 0.46 L for the JTS group compared with 0.04 ± 0.51 L (p < 0.01) for the NS group. (d) The NS group exhibited a significantly greater CoB/CoM distance (d_N_) (2.08 ± 0.40 cm) than the CS group (1.66 ± 0.25 cm) (p < 0.05), and the JTS group exhibited a significantly greater maximum ventilation CoB/CoM distance (dMax) (2.28 ± 0.33 cm) than the CS group (1.78 ± 0.20 cm) (p < 0.01). 10.6084/m9.figshare.2059977.

**Fig 5:**
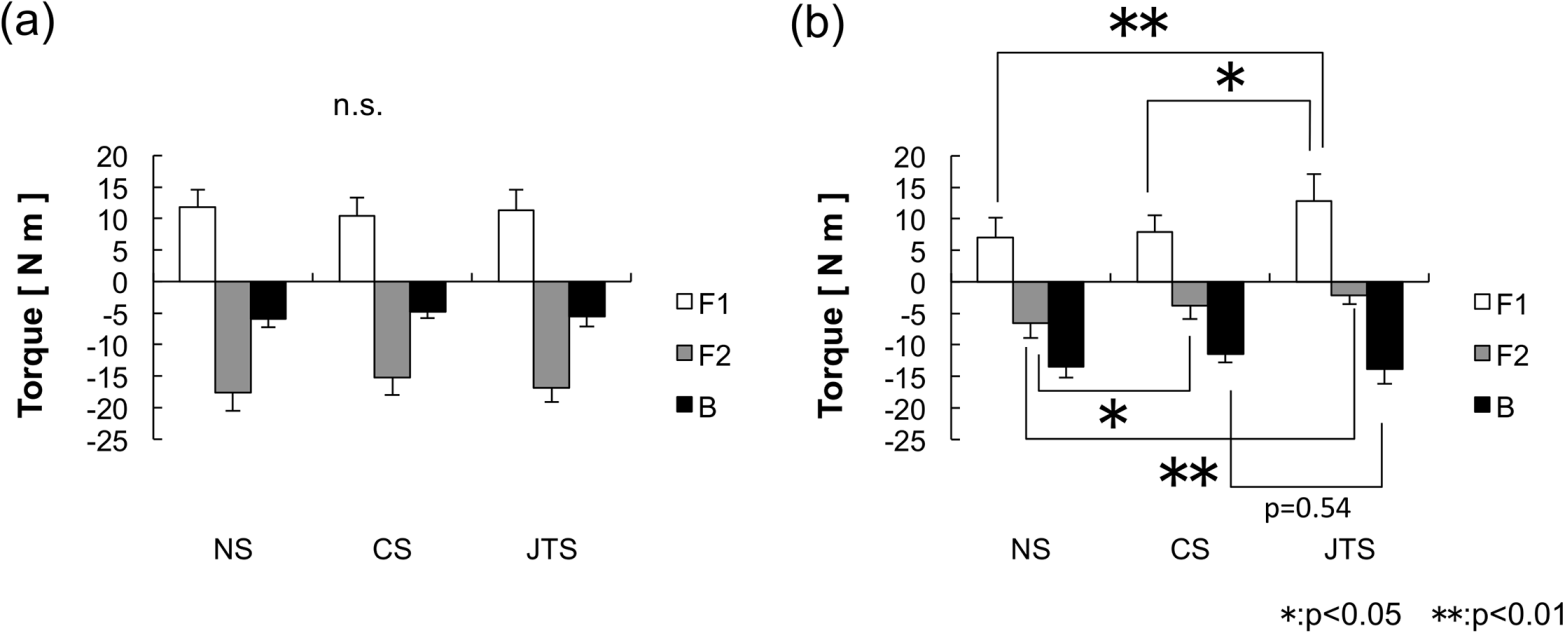
Comparison of upper-limb torque, lower-limb torque, and buoyancy torque at the state of total expiration and maximum ventilation. (a) When total expiration occurs, no differences are observed between each torque’s result (see Fig 5-a). (b) T_F1_ increases at the swimmers’ maximum ventilation as their swimming ability improves; however, T_F2_ decreases (see Fig 5-b). 10.6084/m9.figshare.2059989.

The results of each test subject’s BB ratio, neutral buoyancy’s lung ventilation, maximum ventilation, and CoB/CoM distance are shown in Table 1. The BB ratio results (Fig 4A) show that the JTS group displayed a higher horizontal retention rate than the other groups (JTS group: 0.70 ± 0.02, CS group: 0.65 ± 0.04, NS group: 0.62 ± 0.02); in addition, the JTS group’s BB ratio was significantly higher than that of the NS and CS groups (*F* (2, 23) = 22.708, p < 0.001), as shown in Fig 4A. No difference in lung ventilation was detected among the three groups, either in neutral buoyancy ventilation (LV_N_) or in maximum buoyancy ventilation (LV_Max_) (Fig 4B). The JTS group exhibited significantly greater lung ventilation variation (i.e., the difference between the neutral and maximum ventilation) than the NS group: 0.82 ± 0.46 L for the JTS group compared with 0.04 ± 0.51 L (p < 0.01) for the NS group (see Fig 4C). The NS group exhibited a significantly greater CoB/CoM distance (d_N_) (2.08 ± 0.40 cm) than the CS group (1.66 ± 0.25 cm) (p < 0.05), and the JTS group exhibited a significantly greater maximum ventilation CoB/CoM distance (d_Max_) (2.28 ± 0.33 cm) than the CS group (1.78 ± 0.20 cm) (p < 0.01), as shown in Fig 4D. The average upper-limb torque (T_F1_) at the time of complete expiration was 11.4 ± 3.2 N m for the JTS group, 10.4 ± 2.8 N m for the CS group, and 11.8 ± 2.8 N m for the NS group. The average lower-limb torque (T_F2_) was −16.8 ± 2.3 N m for the JTS group, −15.2 ± 2.8 N m for the CS group, and −17.6 ± 2.9 N m for the NS group; a statistical analysis of these values revealed no statistically significant difference between the three groups (Fig 5A). The average T_F1_ during maximum ventilation was 12.8 ± 4.3 N m for the JTS group, 7.9 ± 2.7 N m for the CS group, and 7.0 ± 3.2 N m for the NS group (F (2, 23) = 6.013, p < 0.01). The average T_F2_ was −2.1 ± 1.5 N m for the JTS group, −3.7 ± 2.1 N m for the CS group, and −6.5 ± 2.4 N m for the NS group (F (2, 23) = 9.463, p < 0.01). Statistical analyses of the T _F1_ and T_F2_ values revealed statistically significant differences between the three groups (Fig 5B).

**Table 1:** Results for each test subject’s Breathing–Balance Ratio, lung ventilation, and CoB/CoM distance

Subject	Group*^1^	Event	BB Ratio	LV_N_*^2^ [L]	d_N_*^3^ [cm]	LV_Max_*^4^ [L]	d_Max_*^5^ [cm]
1	NS	Rugby Football	0.63	2.16	1.10	3.37	1.50
2	NS	Football	0.65	3.35	2.13	3.03	2.01
3	NS	Football	0.59	2.30	2.09	2.20	2.03
4	NS	Football	0.64	1.99	2.29	1.41	2.03
5	NS	Football	0.63	2.79	2.34	2.63	2.27
6	NS	Basketball	0.59	2.48	2.01	2.81	2.15
7	NS	Football	0.63	2.49	2.14	2.50	2.14
8	NS	Badminton	0.62	2.51	2.55	2.43	2.52
9	CS	Butterfly	0.72	1.77	1.54	2.91	1.92
10	CS	Butterfly	0.62	1.72	1.16	2.25	1.37
11	CS	Freestyle	0.65	2.07	1.64	2.59	1.82
12	CS	Freestyle/IM	0.67	2.51	1.47	3.20	1.71
13	CS	Backstroke	0.60	1.81	1.85	2.07	1.99
14	CS	Freestyle	0.63	2.80	1.96	2.80	1.95
15	CS	Butterfly	0.64	2.75	1.73	2.48	1.58
16	CS	Breaststroke/IM	0.64	2.18	1.89	2.26	1.91
17	JTS	Freestyle	0.67	2.77	1.94	3.73	2.38
18	JTS	Freestyle/IM	0.70	2.80	1.92	2.77	1.90
19	JTS	Backstroke/IM	0.74	2.03	2.21	3.38	2.80
20	JTS	Butterfly	0.69	1.61	2.35	2.43	2.80
21	JTS	Breaststroke	0.71	1.94	1.88	3.36	2.51
22	JTS	Butterfly/IM	0.71	1.32	1.90	1.84	2.18
23	JTS	Breaststroke	0.71	2.20	1.59	2.80	1.84
24	JTS	Breaststroke	0.70	1.43	1.70	2.26	2.08
25	JTS	Butterfly	0.68	2.51	1.76	3.90	2.29
26	JTS	IM	0.73	2.09	1.87	2.42	2.02
Mean	NS		0.62	2.51	2.08	2.55	2.08
SD			0.02	0.39	0.40	0.55	0.27
Mean	CS		0.65	2.20	1.66	2.57	1.78
SD			0.04	0.41	0.25	0.36	0.20
Mean	JTS		0.70	2.07	1.91	2.89	2.28
SD			0.02	0.49	0.21	0.64	0.33

*1 NS: Non-swimmer, CS: Collegiate Swimmer, JTS: Junior Top Swimmer.

*2 Lung Volume at Neutral Buoyancy.

*3 CoB/CoM Distance at Neutral Buoyancy.

*4 Maximum Lung Volume.

*5 CoB/CoM Distance at the Maximum Value of Lung Volume.

## Discussion

**Fig 6:**
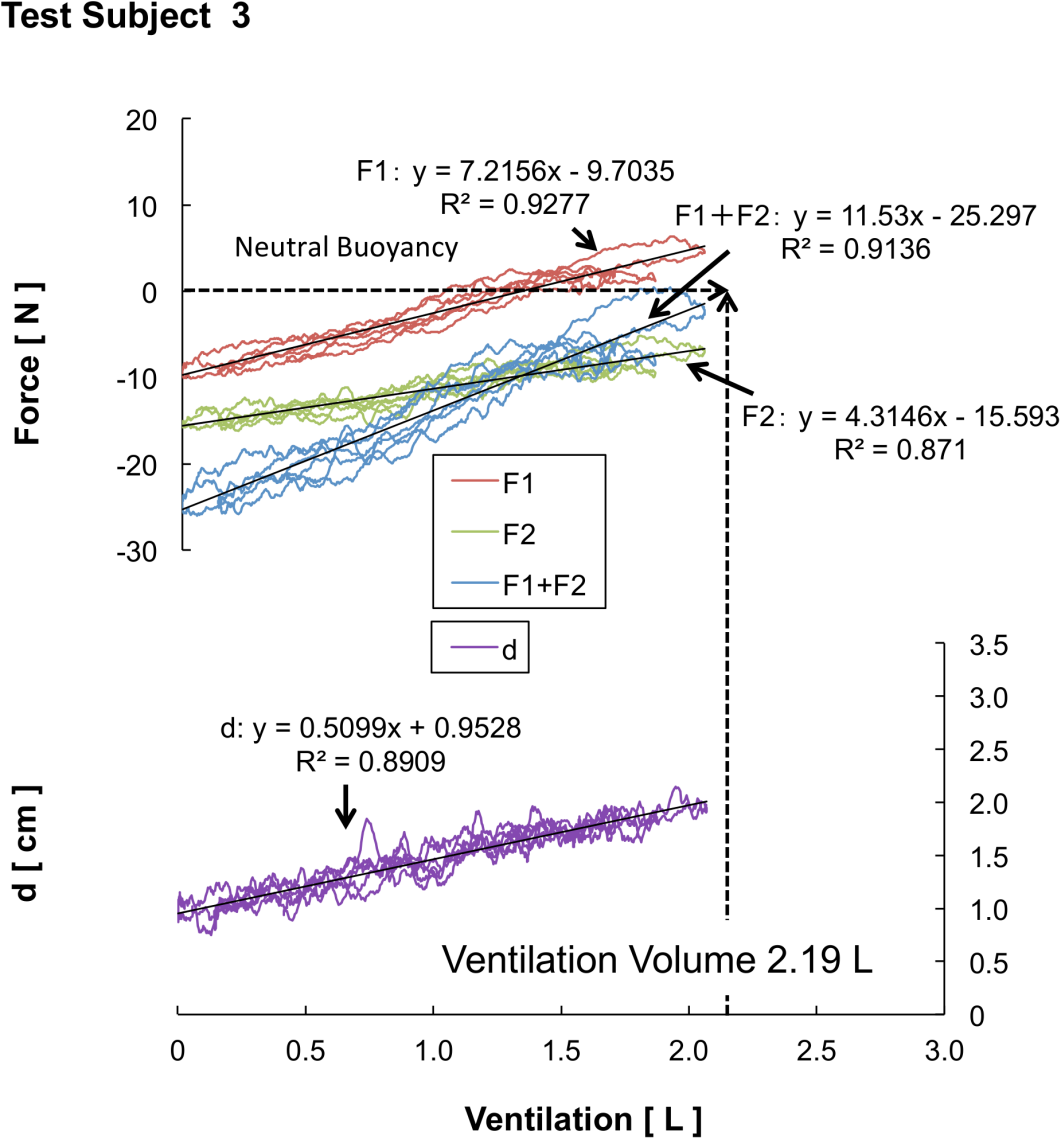
Results of Buoyancy and CoB/CoM distance of Test Subject 3. 10.6084/m9.figshare.2059980.

**Fig 7:**
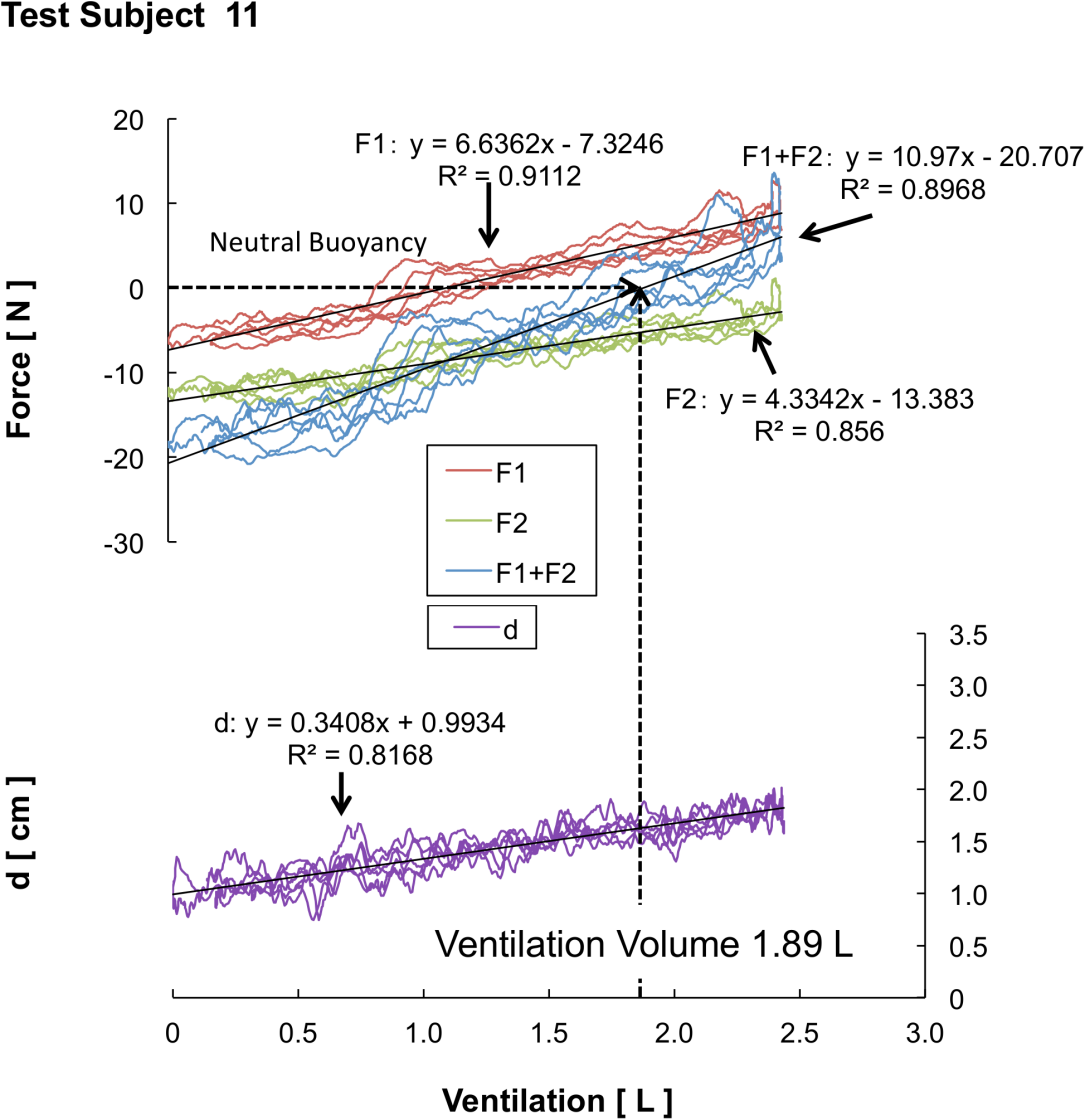
Results of Buoyancy and CoB/CoM distance of Test Subject 11. 10.6084/m9.figshare.2059983.

**Fig 8:**
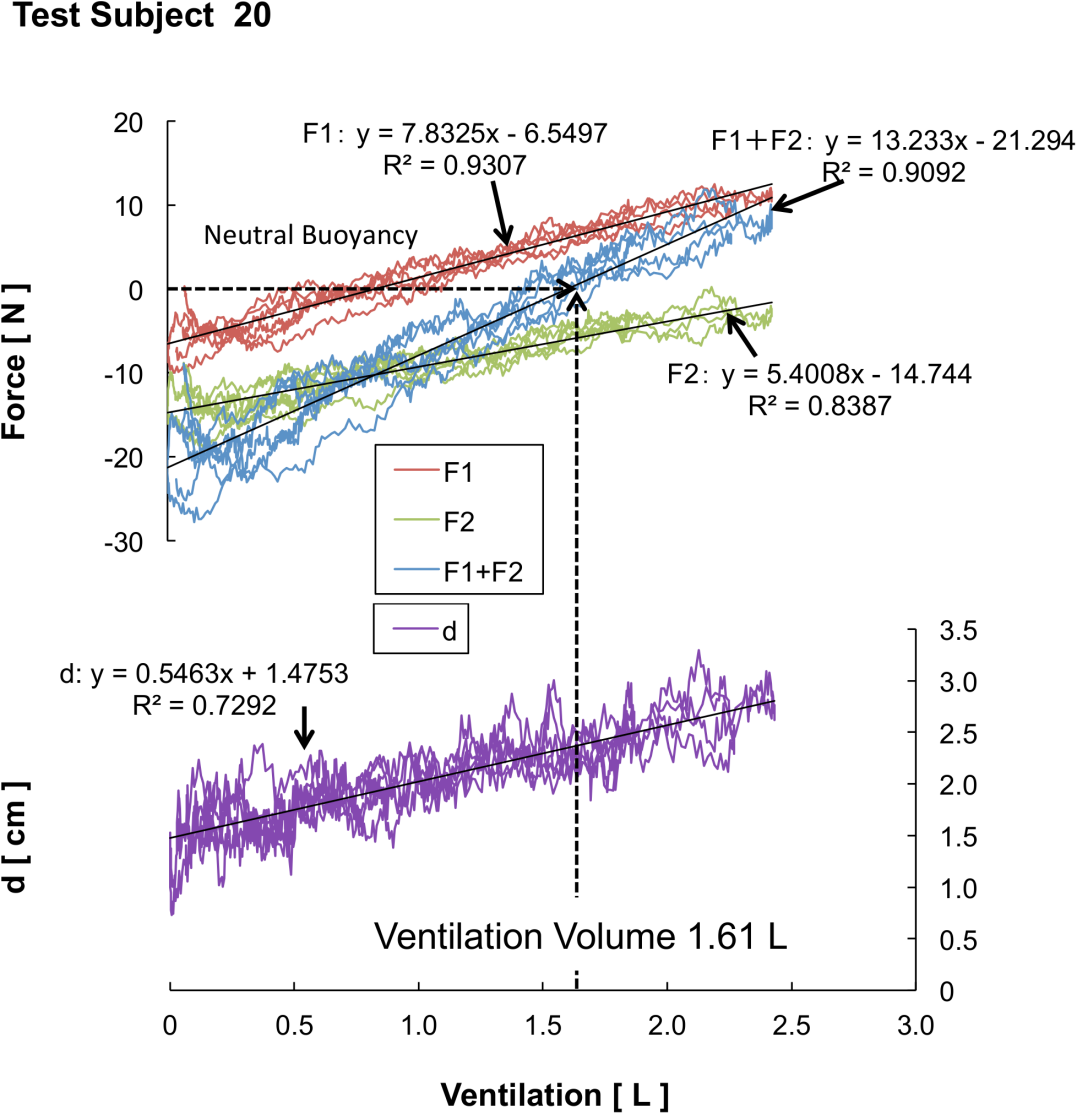
Results of Buoyancy and CoB/CoM distance of Test Subject 20. 10.6084/m9.figshare.2059986.

Based on the BB ratio results and the differences that exist between each of the three test subject groups, we are able to determine the qualities shared by top swimmers. In general, it is considered that the horizontal posture under water correlates with the volume of lung ventilation and magnitude of the buoyancy. However, there was no statistical indication of significant differences in the volume of lung ventilation between the three groups (see Fig 4B). This fact indicates that non-expert swimmers, collegiate-level swimmers, and international competitive junior swimmers exhibit no differences in the volume of lung ventilation. Consequently, the magnitude of buoyancy with inspiration showed no differences among the three groups. It is considered that the amount of change in the volume of lung ventilation and magnitude of the fluctuation of the buoyancy affecting the BB ratio are the major factors influencing the horizontal posture under water. Moreover, it cannot be denied that there is a possibility that the manner of abdominal breathing affects the horizontal posture. Furthermore, Figs 6, 7 and 8 shows an analysis of one trial’s data, which was pulled from just one test subject that was randomly selected from each group. Subject 3 is from the NS group (Fig 6), Subject 11 is from the CS group (Fig 7), and Subject 20 is from the JTS group (Fig 8); these test subject numbers correspond with those given in Table 1. Since the numbers of F1 and F2 load data segments in existence at the time of buoyancy measurements indicate the amount of vertical force at the state of complete expiration, the total of these two forces can be substituted as the test subject’s inner water weight. The average F1 and F2 load data values for each group are as follows: F1: 11.17 ± 2.46 N and F2: 17.36 ± 2.80 N for the NS group; F1: 9.83 ± 2.49 N and F2: 14.61 ± 2.44 N for the CS group; and F1: 10.41 ± 2.98 N and F2: 16.49 ± 2.27 N for the JTS group. Considering that no statistically significant difference exists in these values between the three groups, we conclude that no physical differences in this type exist between the study’s three test subject groups.

The point at which the sum of the vertical forces on F1 and F2 equals 0 is referred to as “neutral buoyancy.” The neutral buoyancy achieved during lung ventilation is considered to be the floating threshold; in other words, the swimmer’s body floats during inspiration and sinks during expiration, and the point between each of these processes, when the body achieves neutral buoyancy, is considered to be the floating threshold. Note that as the swimming ability improves, each swimmer’s flotation threshold shifts to the left side of Figs 6, 7 and 8. Note that Subject 3’s lung ventilation does not go above the flotation threshold even when he/she deeply breathes; thus, this swimmer cannot float up. More than half of the test subjects from the NS group display this same phenomenon. On the contrary, Subject 20’s lung ventilation during neutral buoyancy is 1.61 L, and his/her maximum ventilation is 2.43 L. The average lung ventilation during neutral buoyancy for the JTS group is 2.07 ± 0.49 L, and the average maximum ventilation for that group is 2.89 ± 0.64 L. Since the average variable lung ventilation for the JTS group (i.e., the difference between the JTS group’s average neutral buoyancy ventilation and average maximum ventilation) is approximately 0.8 L, we can conclude that the JTS group effectively utilizes the buoyancy required for upthrusts (see Fig 4C).

In previous studies, a swimmer’s horizontal posture was evaluated according to the positional relation between his/her CoB and CoM (McLean & Hinrichs [15, 17] Watanabe et al. [18]). Therefore, it has generally been considered that shortening the CoB/CoM distance is necessary to achieve the ideal low-resistance posture. The importance of the CoB/CoM distance evaluations cannot be denied. However, as can be observed in Hay’s [9] description, if a “gap” exists between the CoB and CoM in a swimmer’s streamline posture, the evaluation of that swimmer’s posture requires an examination of the influence of the actual torque acting on the swimmer’s body in addition to the conventional examination of his or her CoB/CoM distance.

When total expiration occurs, no differences are observed between each torque’s result (see Fig 5A). Moreover, T_F1_ increases at the swimmers’ maximum ventilation as their swimming ability improves; however, T_F2_ decreases (see Fig 5B). In particular, among the JTS group, the T_F1_ values at total expiration and at the maximum ventilation are the same; the NS and CS groups’ T_F1_ values at the maximum ventilation are significantly lower than those at total expiration. In other words, the JTS group, which excels at swimming, can effectively utilize the buoyancy affecting their chests, thereby making it possible for their lower limbs to float. The mechanism of the floating of the lower limbs can be explained by a third-class lever; the JTS group firmly retains their hands (F1) as the fulcrum of the third-class lever while allowing their feet (which are the point of action) to float to the surface of the water owing to the buoyancy acting during inspiration. Meanwhile, based on their results, we can assume that the NS and CS groups, which have less-advanced swimming abilities, are unable to maintain this fulcrum, leading to their reduced upper-limb torques. If this is indeed the case, then stabilization of this fulcrum significantly involves the shoulder-joint and trunk muscles.

The fact that differences exist in the test subjects’ posture, manner of floating, and CoB/CoM distance, despite there being no variance in the most of the subjects’ physical characteristics such as lung capacity and inspiration-induced buoyancy as well as the variance noted in characteristics such as pitch movement, indicates that it is not buoyancy alone that determines the swimmer’s posture; rather, it is the way in which the swimmer utilizes this buoyancy. Since the shoulder-joint and trunk muscles were not evaluated in our study, we can assume that non-expert swimmers, e.g., the NS group, who are unused to utilizing the muscles around their shoulder joints, lack the same level of glenohumeral joint stability exhibited by competitive swimmers; however, this theory remains a matter of speculation. Konin & Barany [19] state that swimming requires a high level of functional stability and that both the skeletal frame and axial components are necessary for its realization. Moreover, they point out the importance of trunk strength, trunk stability, and glenohumeral-joint range-of-motion control (noting that the range of motion occurring in that joint during swimming is much larger than the average range of motion occurring in that joint during other sports) in achieving the dynamic stroke motions unique to swimming. According to Yanai [6], the majority of a swimmer’s propulsive force is the hydrodynamic force on the hand; this means that the strength and stability of the muscle around the shoulder joints most closely linked to the swimmer’s repetitive stroking motion are among the most important factors determining that swimmer’s performance. Moreover, if the hydrodynamic forces affecting the hand serve as the propulsive force, it is highly possible that the JTS group (which exhibits better performance than the CS group) has superior shoulder-joint strength and trunk muscle stability even though both groups are expert swimmers. In recent years, it has become common for swimmers to engage in exercises designed to strengthen both their trunk and shoulder-joint muscles; it is considered that these exercises increase both the stability and strength of these muscles over the stability and strength observed in the same muscles of athletes engaged in other sports. Lynch et al. [20] reported that after swimmers from an elite national university completed eight weeks of upper-trunk and shoulder-joint muscle training, their scapula functions and postures improved.

Moreover, if the hydrodynamic forces affecting the hands serve as the propulsive force, it is highly possible that the JTS group (which exhibits better performance than the CS group) has superior shoulder-joint strength and trunk-muscle stability even though both groups are expert swimmers. This could explain the differences exhibited in both groups’ functional stability while maintaining a horizontal posture.

In contrast, it should be noted that the buoyancy torque results show a statistically superior tendency between the JTS and CS groups (p = 0.54). In this study, no statistical differences were observed in the physical characteristics, lung ventilation, or buoyancy among the three groups. Thus, any differences existing in the buoyancy torque results must have been caused by differing CoB locations. Buoyancy torque is the product of the buoyant force and CoB/CoM distance. If no difference exists in the buoyancy force, an extended CoB/CoM distance increases the buoyancy torque. In fact, as a whole, the JTS group extended their CoB/CoM distance during the maximum ventilation more efficiently than the CS group (Fig 4D). Conventional evaluations assume that a smaller CoB/CoM distance is more effective. However, the results of this study suggest the possibility that an ideal CoB/CoM distance at which the horizontal posture can be more easily retained exists, partially negating the assertions made in previous conventional reports.

## Conclusions

The international-level junior swimmers surveyed in the present study exhibited statistically significantly higher BB ratio than the other groups of subjects. These results of this study indicate determinate differences in the levelness of the horizontal posture under water even though there are no differences in the physical characteristics and ventilation volume. The BB ratios obtained herein clearly show that there is a difference in the swimmers’ horizontal posture. Thus, it is possible that the manner of abdominal breathing affects the swimmers’ horizontal posture under water. Moreover, it is necessary to determine the appropriate position by balancing the relationship between the CoB and CoM in accordance with the magnitude of torques acting on each part of the body. In conclusion, the BB ratio is considered to be a new indicator that can quantitatively evaluate the horizontal posture of a swimmer. Therefore, the BB ratio may be a useful index for evaluating actual swimming performances and discovering talent.

## Supporting information

S1 Dataset PLOS ONE all relevant data.xlsx. (XLSX)
